# Unpacking sustainabilities in diverse transition contexts: solar photovoltaic and urban mobility experiments in India and Thailand

**DOI:** 10.1007/s11625-017-0438-0

**Published:** 2017-05-17

**Authors:** Rob Raven, Bipashyee Ghosh, Anna Wieczorek, Andy Stirling, Duke Ghosh, Suyash Jolly, Eakanut Karjangtimapron, Sidtinat Prabudhanitisarn, Joyashree Roy, Somporn Sangawongse, Frans Sengers

**Affiliations:** 1 Copernicus Institute, Utrecht University, Utrecht, The Netherlands; 2 Science Policy Research Unit, University of Sussex, Brighton, UK; 3 School of Innovation Sciences, Eindhoven University of Technology, Eindhoven, Netherlands; 4 Global Change Programme, Jadavpur University, Kolkata, India; 5 Sustainable Land Use and Natural Resource Management Program, Chiang Mai University, Chiang Mai, Thailand

**Keywords:** Sustainability, Transitions, Multicriteria mapping, Appraisal, Experiment, Asia

## Abstract

It is generally accepted that the concept of sustainability is not straightforward, but is subject to ongoing ambiguities, uncertainties and contestations. Yet literature on sustainability transitions has so far only engaged in limited ways with the resulting tough questions around what sustainability means, to whom and in which contexts. This paper makes a contribution to this debate by unpacking sustainability in India and Thailand in the context of solar photovoltaic and urban mobility experimentation. Building on a database of sustainability experiments and multicriteria mapping techniques applied in two workshops, the paper concludes that sustainability transition scholarship and associated governance strategies must engage with such questions in at least three important ways. First, there is a need for extreme caution in assuming any objective status for the sustainability of innovations, and for greater reflection on the normative implications of case study choices. Second, sustainability transition scholarship and governance must engage more with the unpacking of uncertainties and diverse possible socio-technical configurations even within (apparently) singular technological fields. Third, sustainability transition scholarship must be more explicit and reflective about the specific geographical contexts within which the sustainability of experimentation is addressed.

## Introduction

Sustainability transitions is a growing field of research (Markard et al. 2012; Chappin and Ligtvoet 2014). This literature argues for sustainability experiments (Sengers et al. 2017; Farrelly and Brown 2011; Kemp et al. 1998) as key alternatives to incumbent, unsustainable systems. Experiments constitute emerging innovation trajectories, which, in turn, can shape broader development pathways (Berkhout et al. 2009, 2010; Rock et al. 2009). As such, experiments are considered instrumental in large-scale transformation of unsustainable systems currently providing human needs such as energy, health or mobility. Such a transformation denotes not only technological but also societal change; hence, the transformation of systems towards sustainability is often referred to as socio-technical systems innovation, or sustainability transition. It is increasingly argued that sustainability experiments may play particularly important roles in ‘emerging economies’ in achieving socio-economic development with minimal adverse impact on the environment (Berkhout et al. 2010; Wieczorek et al. 2015).

The various models developed to better understand the way in which transitions unfold, such as the multi-level perspective (Rip and Kemp 1998; Geels 2002; Smith et al. 2010) make useful contributions to understandings of the process of radical change in general, but they have been criticised for underplaying the evidently political underpinning of sustainable development in general (Scrase and Smith 2009; Kern 2010) and experimentation in particular (Smith and Raven 2012; Raven et al. 2016). The argument is that sustainability is not a neutral exogenous feature in transition processes, simply to be assumed as self-evident or objective. Instead, sustainability is the outcome of negotiations and contestations across plural social interests and involving contrasting power relations in decision-making processes (Walker and Shove 2007; Voss et al. 2006; Leach et al. 2010 McDowall and Eames 2007; Eames and McDowall 2010; Smith 2007; Smith and Stirling 2010; Swilling and Annecke 2012; Newig et al. 2007). Also, at times sustainability is an instrument strategically chosen by decision makers in any highly contested negotiation process. This matters in particular in situations where there is a multitude of innovation options available to decision makers, because they have to decide which options to support in what ways, and which to ignore.

Taking these debates on sustainability and diversity in approaches and motivations seriously has major implications for the governance of sustainability transitions (Smith et al. 2005; Loorbach 2010). Rather than simply assuming a priori some technological options to be sustainable, or seeking single objective rankings of ‘the best’, ‘most sustainable’ or ‘economically most efficient’ technological solutions, transition governance requires decision making in the context of multiple, often diverging appraisals of socio-technical options (Stirling 2011; Smith and Stirling 2007).

It is quite a challenge to those involved in decision making for governing sustainability transitions, and thus for those involved in niche experimentation. For decision makers, the challenge is how to decide which options to support given the legitimate need for economic development that is socially just and within ecological safe limits? Indeed, a popular perception is that economic development and ecological impacts are in conflict and future developmental choices are uncertain. How can we orchestrate fair decision making on these issues in the light of political economies that prioritise some options over others?

This paper aims to make a contribution to this debate through analysis of the diverse ways in which different actors in different contexts appraise sustainability of niche experiments. The empirical analysis covers two national contexts (India and Thailand) and two sectoral contexts fields (solar photovoltaic (solar PV) and urban mobility). The paper develops a pragmatic framework to map a number of different kinds of diversity relating to sustainability transitions—in terms of the performance of experiments, the appraisal of these experiments by different social groups and individuals, the different sectors and the different national contexts. The research question is formulated as follows: How are emerging innovation trajectories for solar PV and urban mobility appraised by various actors under different perspectives in India and Thailand?

The paper is structured as follows. Section 2 discusses the relevance of diversity in sustainability transitions as well as the analytical approach in this paper. Section 3 discusses the background and methods for our empirical work. Section 4 presents results. Section 5 discusses and concludes.

## Diversity and governance in sustainability transitions

Diversity is an important facet in the governance of sustainability transitions (Stirling 2011; White and Stirling 2013). Diversity in possible options/trajectories for sustainability transition can offer benefits in the capacity to adapt in the face of future uncertainties and unexpected developments. By avoiding ‘betting on one horse’, but instead maintaining multiple differing socio-technical variants, a given socio-technical system can improve its capacity to deal with future shocks. Diversity is also seen to matter in terms of improving competition and shaping effective innovation processes, as well as in developing socio-technical systems in such a way that they are better tailored to the variety of spatial conditions present in different regions, communities, countries or other kinds of contexts. Finally, diversity is also argued to be important in sustainability transitions as a way to navigate—and potentially accommodate—complex and plural social and economic interests that may be irreconcilable in other ways (Stirling 2010).

Maintaining a balanced variety of disparate innovation options is thus considered an important condition in the governance of sustainability transitions. Yet the notion of sustainability itself is not a straightforward concept, but subject to ongoing ambiguities, uncertainties and contestations (Voss et al. 2007; Meadowcroft 2007; Hugé et al. 2013; Stirling 2010). This presents decision makers engaged to decide which innovations to support (or not) with a challenge, because there are no universally supported environmental, economic and social sustainability goals that can apply to any given context. Such aims and priorities are deeply connected to contrasting cognitive understandings, value positions and social interests (Shove and Walker 2007). Limited knowledge and uncertainty about future relationships between society, technology and nature also complicate present decision making about which niche experiments to support and which not. What may be perceived as appropriate at some point in time within a given, but inherently limited set of knowledge about future environmental, social and economic implications, may turn out to be rather unsustainable when new relationships and implications are later on uncovered. The above implies that the governance of sustainability transitions is a deeply political and complex, controversial process, because appraisal of the diverse range of innovations is not a straightforward, singular process. Social learning and engagement of diverse groups in society in decision-making processes is, therefore, crucial in these processes.

Very few studies have paused to investigate empirically the diversity in sustainability transitions articulated around various experiments competing for political and societal attention and resources, across a diverse set of sectoral and geographical contexts. Smith (2007), for instance, has demonstrated how the meanings and understandings of what is considered sustainable changed and diversified when eco-housing and organic farming experimentation grew out of their initial grassroots niches to become part of incumbent socio-technical regimes. A notable exception is also provided by Eames and McDowall (2010) and McDowall and Eames (2007). Building upon pioneering work by Truffer et al. (2008), they report on experiences with a comprehensive approach using visioning techniques, workshops and multicriteria mapping, to envision and assess various pathways towards a hydrogen economy. More generally, the current paper is positioned in sustainability assessment literature, for which different analytical frameworks have been proposed, and critically reviewed in earlier work (Stirling 1999, 2006).^1^

It is against this background that the ambition and main contribution of this paper are to contribute to this lively debate by unpacking empirically how the notion of sustainability is perceived in the context of experimentation in developing countries. Our approach is similar to that developed by Eames and McDowall (2010), though different in some of its theoretical assumptions.^2^ Moreover, the work is situated in emerging Asian economies, with a particular focus on solar PV and urban mobility experimentation in India and Thailand, whilst the existing transitions work is mostly located in well-developed economies. (Berkhout et al. 2010; Wieczorek et al. 2015). These cases and countries were chosen, because solar PV and sustainable urban mobility are receiving major policy attention in both the countries, whilst India and Thailand represent both a lower middle-income and an upper middle-income country, respectively.

As a starting point this paper develops a pragmatic framework for unpacking the diverse ways in which various actors perceive sustainability across various sets of experiments (to which we will refer as ‘experimental trajectories’), and in the context of different national and sectoral contexts. This framework resulted from iterative analyses and comparing results, and rests in mapping diversity in the following dimensions:

- Performance diversity: diversity in terms of sustainability performance observable across a variety of experimental trajectories in the focal field. This diversity aims to offer the starting point for analysis and allows us to unpack further forms of diversity in terms of socio-political perspectives, geographical locales and sectoral contexts.
- Appraisal diversity: diversity in terms of divergent understandings and priorities in appraisal and associated differences in patterns of performance as appraised under different relevant perspectives. This aspect of diversity allows us to unpack how different actors use different kinds of criteria for assessing sustainability, with different levels of uncertainty, and how they frame different kinds of priorities in the ranking of experimental trajectories.
- Sectoral diversity: diversity in the nature of sustainability appraisal as applied across contrasting socio-technical systems, in this case solar PV and urban mobility. This aspect of diversity allows us to unpack differences in the kinds of appraisal criteria used in different sectoral contexts and explore associated implications for different notions of sustainability.
- Geographical diversity: diversities in the character of appraisal and associated rankings as between different spatial contexts, in this case national situations. This aspect of diversity allows us to unpack how different arrays of criteria are used in different national contexts and how these shape different pictures of performance rankings.

In the following section, we discuss the methods used to address these central objects of interest.

## Methods

The methodological approach for exploring sustainability in Asian experiments involved a threefold process: the construction of a database of sustainability experiments, multiple stakeholder workshops in India and Thailand, and the use of the multicriteria mapping (MCM) software.

### Database

The first methodological step concerns the identification of the range of sustainability experiments with solar PV and urban mobility that are taking place in India and Thailand, brought together in an excel database. The database contains factual information about experiments such as the location, start/end date and triggers; actors involved and outcomes of the experiments. Data were collected for initiatives started in the period 2000–2012 based on online search of websites and databases maintained by: governmental actors,^3^ industry, knowledge institutes (identified through a Scopus search on relevant publications), domestic and international NGOs, international organisations. Some websites provided existing overviews of projects. In other cases, we browsed organisational websites for relevant data or used search-boxes on the websites to find project descriptions. This search results were initially grouped by the research team into ‘experimental trajectories’ (categorising similar experiments into aggregate groups) and provided insight into the historical evolution of solar PV and alternative urban mobility in both countries (see Table 1). The initial grouping was discussed with stakeholders during the workshops, which mostly confirmed the initial grouping exercise. The groupings were used as a starting point for the multicriteria mapping analysis in step 3. Database construction, including a discussion of its methodological details and limitations, is described in detail in Wieczorek et al. (2015).

**Table 1:** Experimental trajectories in solar PV and urban mobility in India and Thailand

	India	Thailand
Solar PV	Solar lanterns	Off-grid generation systems
	Solar home systems	Solar home systems
	Micro-grids	Mini-grids
	Rooftop solar	Rooftop solar
	Solar power plants	Solar power plants
	Solar city	
Urban mobility	Walking	Cycling and walking
	Cycling	Shared transport (shared bikes, cars, songthaew^a^)
	Alternative public transport (bus rapid transit/BRT)	Alternative public transport (BRT, mass rapid transit/MRT, monorail)
	Electric vehicles	Electric vehicles
	Alternatively fuelled vehicles (CNG)	Alternatively fuelled vehicles: (ethanol, CNG, hybrid, solar)
	Vehicle parts innovation	

The names and groupings of the experiments and trajectories differ in the two countries, because they are identified through inductive, bottom-up analysis, taking into account local specificities

^a^A songthaew is a shared transport vehicle in Thailand, also known as ‘red trucks’

### Stakeholder workshops

Despite the general agreement that sustainable technologies, such as solar PV or ‘Bus Rapid Transit systems’ (BRT), have the potential to make a significant contribution to sustainable development policy goals, the future of these technologies is often contested while the views on the meaning of sustainability are potentially conflicting (e.g. McDowall and Eames 2007). To complement this systematic search for experiments, we organised a consultation process with a number of solar PV (17 in India, 15 in Thailand) and urban mobility experts (12 in India, 17 in Thailand) in stakeholder workshops that took place in November 2013 in Kolkata and in May 2014 in Chiang Mai, and which were part of a larger research project on sustainability experimentation in India and Thailand.^4^ The workshops were composed predominantly of stakeholders from each nation. The selection of participants was grounded in in-depth understanding of each case, and developed relationships, as part of a 4-year international research project. Different perspectives in relation to professional background of the participants have been considered, such as a governance perspective (comprising people from ministries, local or regional governmental bodies), academic perspectives (comprising professors, researchers associated with a university or an independent research institute) and industry perspectives (comprising people representing a private firm or organisation) (Table 2). Participation was entirely voluntarily and those who attended came to participate based on their own interest and decision. Participants did not receive a fee, but payments for travel and hotel were made. Participants received a report with project results afterwards. Many of those who attended articulated that an important benefit from participation was exposure to new knowledge and new methods for research. They got introduced to a new overarching framing concept for assessing sustainability experiments, and realised that there is wide diversity in understanding of the concept of sustainability. Participating foreign nationals were not engaged in the appraisal of options but facilitated the workshop and explained the framework.

**Table 2:** Perspectives and individuals in each sector and country

Country	Sector	Perspectives	Number of individuals
India	Solar PV	Academics	9
		Governance	2
		NGO	2
		Industry	2
		Consultancy	2
	Urban mobility	Academics	8
		Governance	2
		NGO	2
Thailand	Solar PV	Academics	8
		Governance	3
		Industry	4
	Urban mobility	Academics	8
		Governance	2
		Consultancy	5
		NGO	2

Academics refer to individuals working at universities. Governance refers to individuals working in public policy institutes and those closely related to public policy decision making. NGO refers to individuals working in non-governmental organisations. Industry refers to individuals working in industrial organisations related to the field. Consultancy refers to individuals working in technical consultancy organisations

The stakeholders with experience through long years of engagement in these domains were invited to present their perspectives on each of the emerging trajectories in interactive plenary sessions, which provided participants with an initial understanding of each of the trajectories. In sector-specific intensive workshops, each of the relevant stakeholders appraised the trajectories according to their own notions of sustainability and understandings of the performance of the different trajectories. Although this cannot be claimed as a definitively robust and representative sample of all relevant views, such a concept is in any case intrinsically problematic (O’Neill 2001). What is more relevant to the mapping of diversities is confident coverage of an envelope of perspectives in the key relevant dimensions (Coburn and Stirling 2016). The present disparity of perspectives was certainly sufficient for the purpose of our key interest in exploring the existence and relevance of diversity in apprising sustainability. In particular, the elicited diversity provided ample substantiation of the central aim of demonstrating the relevance of a great diversity of views on sustainability experiments. If the range of stakeholders engaged in the present study can be regarded as somewhat narrow, then it follows that a more wide-ranging recruitment process would correspondingly have yielded an even greater degree of diversity.

### Use of MCM software

The method used to ensure systematic and symmetrical attention across diverse trajectories and perspectives was a novel hybrid quantitative/qualitative web-based software tool called multicriteria mapping (MCM). For more detailed descriptions of MCM, we refer to previously published work (for details see Stirling 1999; Stirling and Mayer 2000, 2001; Stirling et al. 2006; Burgess et al. 2007; McDowall and Eames 2007; Eames and McDowall 2010; Coburn and Stirling 2016).^5^ In short, MCM is concerned to help ‘broaden out’ and ‘open up’ societal debates about political choices through: (1) a systematic articulation of all relevant perspectives (on, for instance, new technologies); (2) illuminate the range of uncertainties within and ambiguities between each of these perspectives; and (3) document qualitative data concerning the reasons and arguments constituting these perspectives and uncertainties. Hence, MCM has the particular feature that it focuses equally on quantitative representations of performance under different perspectives, at the same time as documenting qualitative information concerning the reasons for performance patterns and uncertainties under each perspective.

**Fig. 1:**
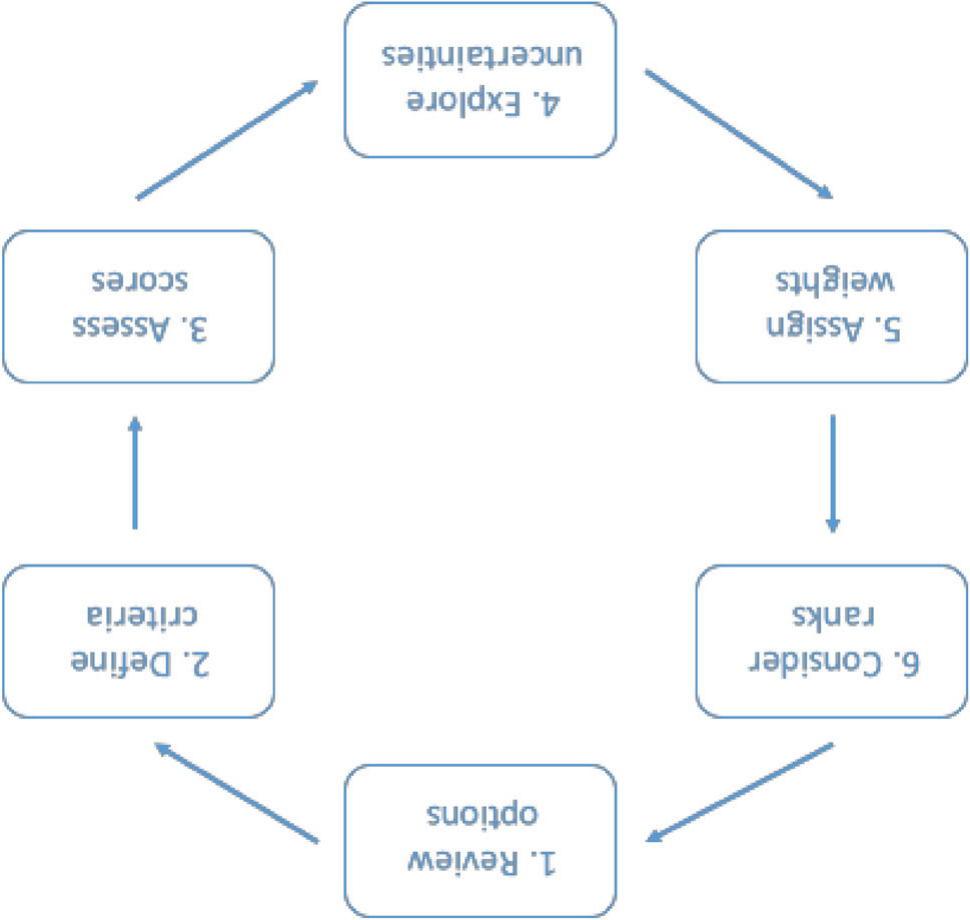
The multicriteria mapping process

In a group workshop providing both for collective deliberation and individual appraisals, trained facilitators recorded through a series of steps, a diversity of stakeholder perspectives. Together these permitted collection of relevant qualitative and quantitative data concerning: the framing and constituting of technological options and their contexts; contrasting ways to conceive and evaluate notions of sustainability itself; divergent understandings and associated uncertainties with regard to the sustainability performance of specific technologies; and individual and collective rankings of the experimental trajectories. The data gathered, therefore, encompassed a deep and wide diversity of issues, including insights concerning the most salient factors distinguishing a plurality of interpretations and positions taken in the sustainability debate around specific innovations. As a result, MCM allowed the mapping of many key sensitivities concerning the performance of particular trajectories as seen under different relevant perspectives, along with details concerning associated uncertainties and framing assumptions. Figure 1 summarises this process.

To ensure the necessary basic common understanding of the exercise as a whole, the project team first introduced the participants to the prior characterisations of the 6–7 experimental trajectories (in MCM, these are referred to as ‘options’; we use these terms here interchangeably). In reviewing these options, participants could also introduce into their own appraisal any relevant variants or wider possibilities that had not been pre-defined. But in this exercise, these ‘additional options’ were not systematically appraised by all other participants.

Second, in the define criteria stage, participants were informed of a literature survey concerning relevant sustainability criteria. This was simply to prompt thinking about the kinds of issues as they might each see them, in order to address what might otherwise be concerns on the part of participants over the need for supporting information. However, there is no necessity on grounds of rigour or consistency in such a process, for participants all to use the same criteria scheme or data. So full flexibility was afforded to participants to formulate whatever they considered under their own perspective to constitute the most relevant sustainability criteria for the context in hand. This was informed by the common data where participants wished, but they could also depart from this common data set where they felt appropriate, in which case qualitative reasons for such departures were documented during appraisal. To this end, facilitators ensured that participants described exactly what they meant by each criterion and why.

Third, in the assess scores stage, participants were again able to consult background data provided by the research team to inform their own understandings where they wished, concerning the performance of each option under each criterion. But participants were again not forced simply to adopt the provided data but were instead free to express divergences—with associated reasons again being documented by facilitators. This process involved assigning scores on an arbitrary ascending interval scale from low to high performance with respect to each criterion. Participants could use any scale they felt comfortable with (typically 1–10 or 1–100)—but this could vary without incurring comparability problems. The software converted raw scores to normalised intervals and it is these relative orderings that are the subject of comparison, not the absolute values of the raw scores.

As part of this scoring process, a fourth feature of the MCM appraisal was that participants were encouraged to pay due attention to any uncertainties they might hold concerning possible differences between optimistic and pessimistic futures of the options. This meant assigning two scores for each option under each criterion: the first on the basis of reasonable assumptions that they feel would yield the most optimistic outcome, the second relates to reasonable assumptions under which a pessimistic outcome might be anticipated for the chosen option. If a participant experienced no uncertainty, these scores could be the same. This unusual feature of MCM captures the degree of fine-grain uncertainty and variability associated with particular features of the performance of specific experimental trajectories under a given criterion. Participants were again asked to talk about the assumptions lying behind these different scores, and these qualitative data were transcribed. In a relatively efficient way, this captured the effects of uncertainty concerning how sustainable an option might prove in practice in any given setting, variability with respect to contrasting possible settings, and sensitivity to wider contextual conditions underlying all settings together.

Fifth, in the assign weights stage, participants were asked to assign simple numerical weights to express the relative importance (in their own view) of each criterion in comparison to others. Prompted by the software, what this involved was the comparing of relative importance for each participant, of the difference between best and worst performance under each criterion. This task was undertaken interactively, informed by the consequent changes in the overall resulting rankings as any weighting changes were applied. Discussion was again documented for comparison with appraisals of other actors.

In the sixth and final stage of the MCM appraisal, participants were asked to consider ranks. This involved each participant examining in detail the visual representation of the overall rankings of the different experimental trajectories—according to their own criteria, scores, uncertainties and weightings. The software calculated these ranks based on a standard ‘linear additive weighting’ procedure, with appropriate normalisation of each score and weighting scale. This enables the ranking patterns of different participants to be compared in terms of their contrasting normalised intervals. No appraisal was regarded as complete, until the participant in question had expressed themselves to facilitators to be positively satisfied with their own ranking pattern as a reasonable expression of their own view concerning the relative performance of the different options.

**Fig. 2:**
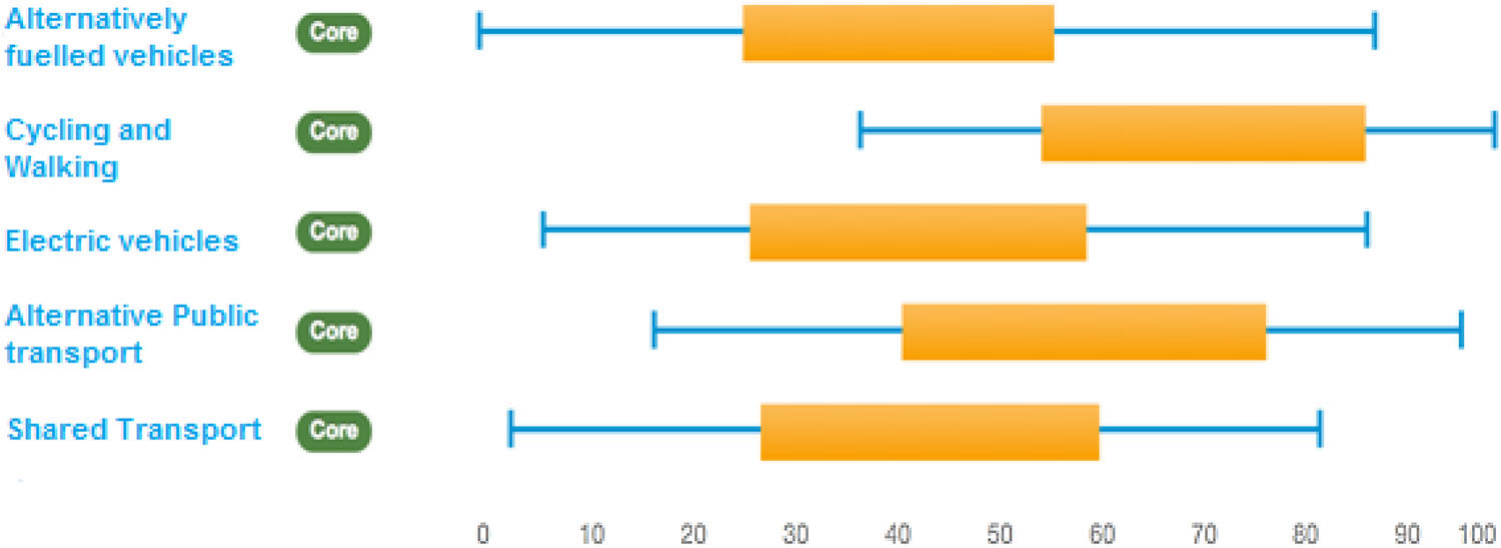
Performance diversity for the Thai urban mobility case

In the resulting ranking charts (see, e.g. Fig. 2), the thin blue lines represent the range between extrema defined between the lowest pessimistic rank and the highest optimistic rank obtained by any participant for each experimental trajectory. The thicker orange bars represent the difference between the mean pessimistic rank and the average optimistic rank across all participants for a given trajectory. So, the right end of each bar represents performance of that trajectory under high optimistic scores on average, while the left end of the bar represents average performance of that trajectory under pessimistic scores. In general, the further the bars and lines extend to the right the more sustainable the experimental trajectory is considered to be.

It is crucial to this process as a means to map divergent perspectives that participants could see the overall patterns of performance and uncertainty derived for the different options in their appraisal and were actively invited to reflect on whether this conformed to their initial expectations and feelings. If not, participants could make a back-up of the original appraisal, and explore other weighting schemes, or revisit their criteria and scoring. Where any such changes were made, facilitators would enquire and document the associated reasons. Only in this way can there be confidence that results authentically reflect the perspectives of different participants, rather than serving to ‘fix’ these through the initial framings imposed by researchers or contingent features of the analysis. The attention to documenting reasons at every stage also provides a means to guard against strategic behaviour on the part of participants themselves.

After the workshops, the research team analysed the qualitative and quantitative data collected. Initial reading and interpretation resulted in two kinds of groupings, each iteratively explored during analysis. First, we grouped individuals in different ways, such that they reflect different notions of what might constitute relevant social perspectives. Second, we grouped criteria in different ways, to explore contrasting ways to divide up sustainability issues across social, economic, environmental and technical factors. Experimental trajectories themselves could also be grouped in different ways if wished. With each permutation of groupings in analysis, associated qualitative descriptions provided in the stakeholder interviews were clearly displayed by the software, in order to ascertain the associated kinds of reasoning in each case. This proved especially helpful in addressing ambiguities between different perspectives in interpreting different criteria. In the case of Thailand, for instance, we added a fifth group of criteria—policy—because Thai stakeholders placed much stronger emphasis on these than did the Indian participants. This facility in MCM to integrate qualitative and quantitative factors in analysing contrasting groupings of key parameters assisted in meaningful interpretations of diversities across perspectives, sectors and geographies.

In the next sections, we present the results of our analysis. Given the vast amount of available material, we opted not for a complete presentation of all results, but decided to highlight the most salient results in relation to the aim of this paper, which is to show diversity in appraisals of sustainability of socio-technical options.

## Results

### Performance diversity

Performance diversity refers to the differences that can be observed in the overall sustainability performance for each of the solar PV and urban mobility trajectories in the two countries. Aggregating the appraisals of all individual stakeholders, this diversity is expressed as contrasting ranking intervals compared across the different socio-technical options within each sector. Figure 2 illustrates this for the Thai urban mobility case.

In this figure, the overlaps in the ranges for different trajectories show (as is typical in MCM) the combined effect of a high degree of uncertainty, ambiguity and variability in the performance orderings of different options. This uncertainty is typically understated in other kinds of appraisal method. This said, it might cautiously be observed that non-motorised transport (such as walking and cycling) received relatively high optimistic scores on average, compared to all other trajectories. A majority of stakeholders in this case agreed that non-motorised transport is the most desirable, as they perceived that they are flexible, affordable and least harmful to the environment. One of the Thai stakeholders adds “…in addition, walking and cycling provide better and easier access to the small alleys (soi) in Thai cities.” This strong preference is reflected in a relatively high mean ranking for the non-motorised trajectories (the mean ranking being the midpoint of the thick orange bars).

The mean ranking for the alternative public transport trajectory is also quite high. Despite the carefully documented differences, stakeholders agreed that alternative public transport systems such as bus rapid transit are in general more inclusive, provide better accessibility and minimise congestion. Where a method (like MCM) avoids forcing closure in appraisal, the emergence of such convergence is correspondingly more robust. Likewise, both cycling and walking as well as alternative public transport trajectories tend to be perceived in general as more sustainable options than the alternatively fuelled vehicle such as CNG cars, electric vehicles and shared transport trajectories in Thailand. This result might be thought significant in relation to frequent patterns of emphasis in innovation for sustainable urban transport.

Interestingly, the mobility trajectories that appear most sustainable are those which are less dependent on high-tech innovations, are more reliant on behavioural shifts and are compatible with existing infrastructure. Alternatively, fuelled vehicles and electric vehicles received considerably lower ranks, as the stakeholders argued that these require high initial investment; they are non-affordable by the poor and middle-income groups (that constitutes a large section of the population) and, therefore, are non-inclusive in nature. Shared transport systems mainly received pessimistic scores due to their current non-environment friendly fuel use and a substantial role in creating congestion, air and noise pollution in the cities of Thailand.

**Fig. 3:**
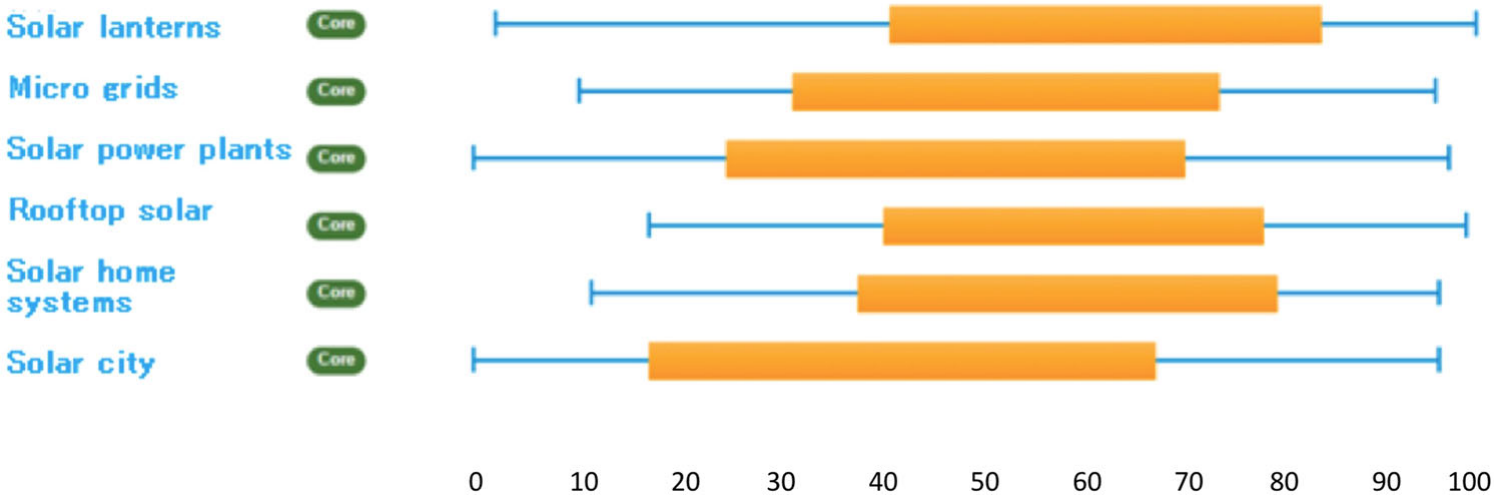
Performance diversity in Indian solar PV case

Analysing performance diversity for solar PV trajectories in India, we observed even more pronounced uncertainties, ambiguities and variabilities in final rankings—measured through high ranges of optimistic and pessimistic scores resulting in substantial overlaps in the sustainability performances of the various trajectories. Figure 3 (below) illustrates this.

This is despite the fact that the different trajectories in this case involve much more similar kinds of technologies than in the transport sector (since all in this case involve photovoltaic cells). One finding in this respect is that decentralised solar PV options like solar lanterns, solar home systems (SHS), and rooftop solar applications seem to display somewhat higher sustainability performance in terms of highest average ranks, compared to centralised large scale solar PV applications like large grid connected solar power plants and solar cities. This picture is revealed by the qualitative discussions of participants during appraisal, where it was argued that decentralised options tend to display advantages over large-scale systems in terms of cost minimisation, easy and quick installation, and operation and maintenance facilities. An Indian stakeholder summed it up in her comments during the interview process “…these small scale individual household based applications will have maximum positive social, economic and environmental impact through maximum accessibility to remote areas deprived of electricity, minimum emission and transmission losses and everything, assuming the subsidies continue for some time; the production and disposal of the equipment are hazard-less.”

Overall, these findings urge caution over more simplistic accounts of the sustainability performance of the different innovation trajectories in either solar PV or mobility. The picture is not straightforward and depends on highly specific visions of what is meant by sustainability. In itself, this holds important implications for notions of transitions and experimentation in which sustainability or its technological implications are held to be self-evident. A key implication is that manifestly divergent informed opinions led to quite extreme ranges in scoring. This underscores the importance of uncertainties in individual perspectives, ambiguities across contrasting perspectives and variabilities across different contextual condition that can often be missed in appraisal. This understanding leads us to the next dimension of diversity, namely appraisal diversity.

### Appraisal diversity

Appraisal diversity is defined as contrasts in understandings, perceptions and values as between different stakeholders participating in the appraisal process. These divergent perspectives on the meanings of ‘sustainability’ were reflected in participants’ selection of criteria, the ways in which these criteria are weighted, divergent patterns of scoring and expressions of uncertainties under individual criteria. This appraisal diversity can be captured by comparing the responses of the stakeholders either at an individual level or at a semi-aggregated level where each of a number of variously definable groupings of stakeholder perspectives can be compared with each other.

**Fig. 4:**
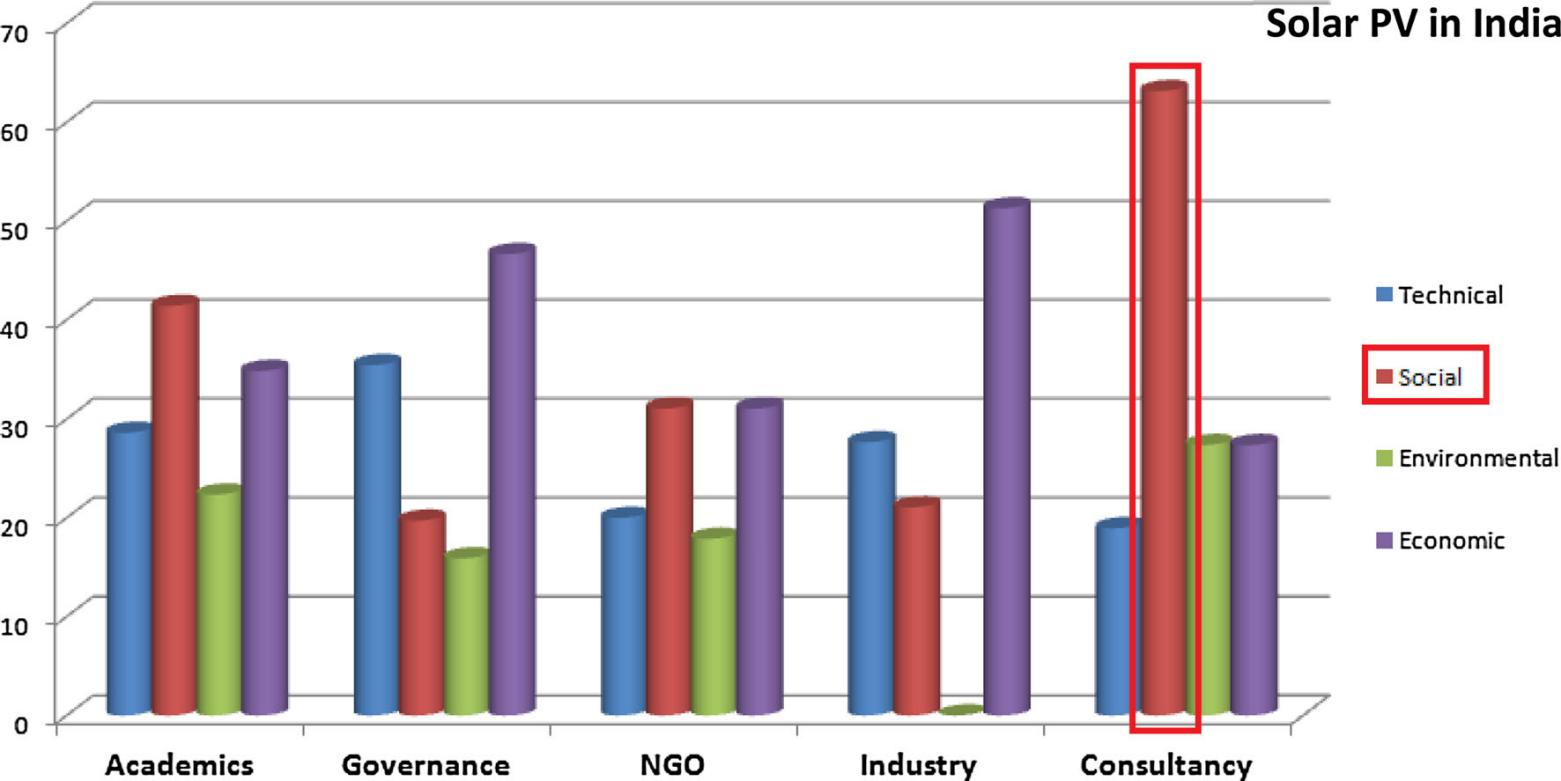
Appraisal diversity in assigning weights to each group of criteria for solar PV case in India

As an example of this analysis at a semi-aggregated level, we compared the weights assigned to each group of criteria (technical, social, environmental and economic) under stakeholder perspectives disaggregated across ‘consultancy’, ‘Industry’, ‘NGO’, ‘governance’ and ‘academics’). Results from the solar PV case in India are shown in Fig. 4.

It is noteworthy that the individuals identified on the basis of their affiliations as consultants assigned strikingly higher weights to social criteria in the appraisal of solar PV in India. They emphasised the importance of local skill development for decentralised maintenance and operation of the solar PV systems, need for supportive policy targeted towards the benefit of “common people”. Another interesting result in Fig. 4 is that the individuals identified as industry actors assigned almost negligible weight to the environmental aspects of sustainability. In expressing their own framings of sustainability criteria, these actors in India emphasised the more socio-economic ‘sustainability’ criteria (like policy and awareness, value of stakeholders, profitability, affordability and entrepreneurship opportunities).

**Fig. 5:**
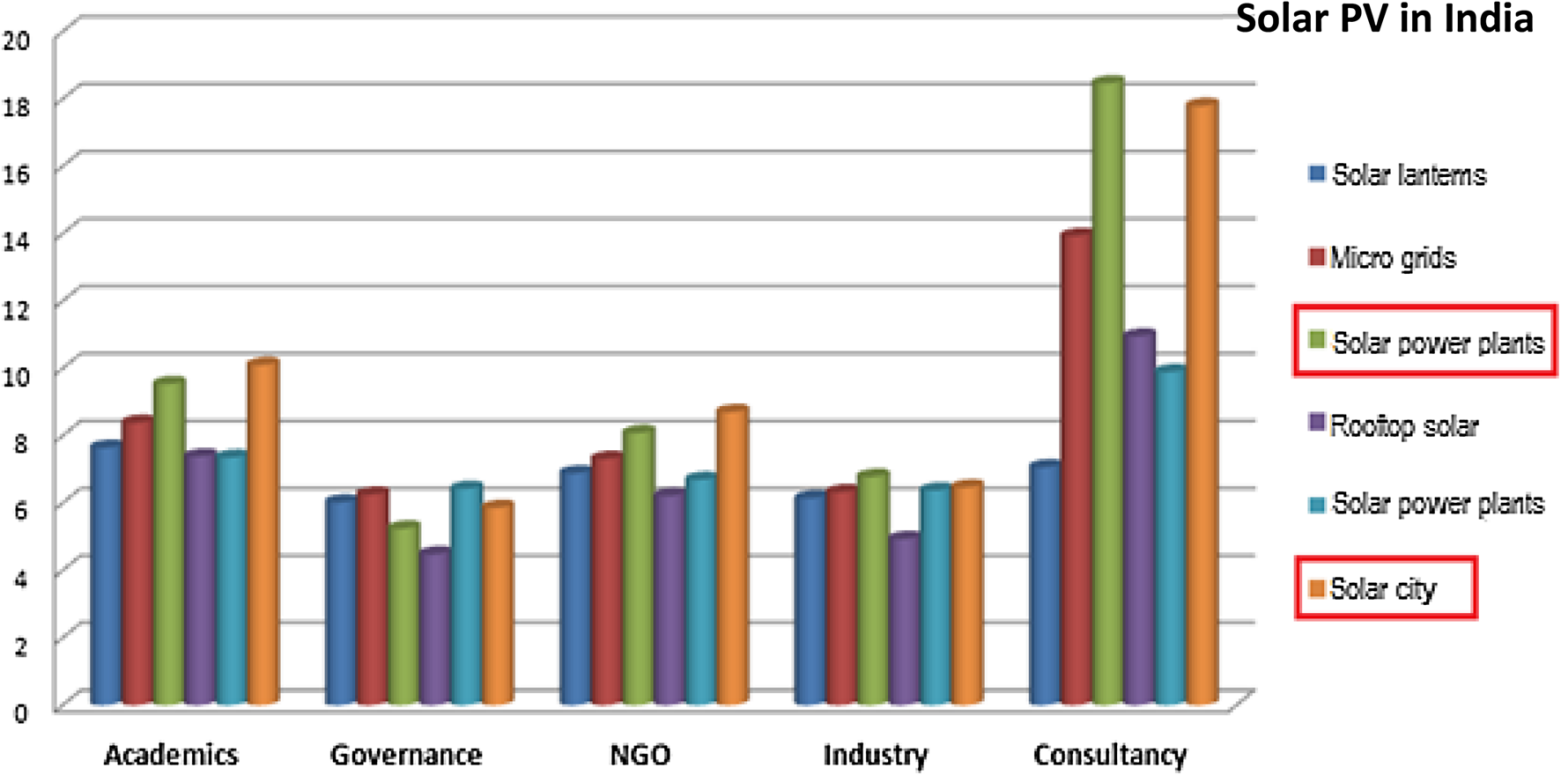
Appraisal diversity in expressing uncertainties for each solar PV trajectory in India

The consultants also expressed significantly higher levels of uncertainty for the centralised solar PV options like power plants and solar cities. Technical consultants tended to take into account the subsequent risks of the systems being highly subsidy dependent, and the policy strategies and financial schemes being less transparent and heavily subject to corrupt practices. An illustration of this point can be quite clearly seen in Fig. 5 (below), where the green and orange bars representing solar power plant and solar city trajectories, respectively, are manifestly tallest for the consultancy perspective.

**Fig. 6:**
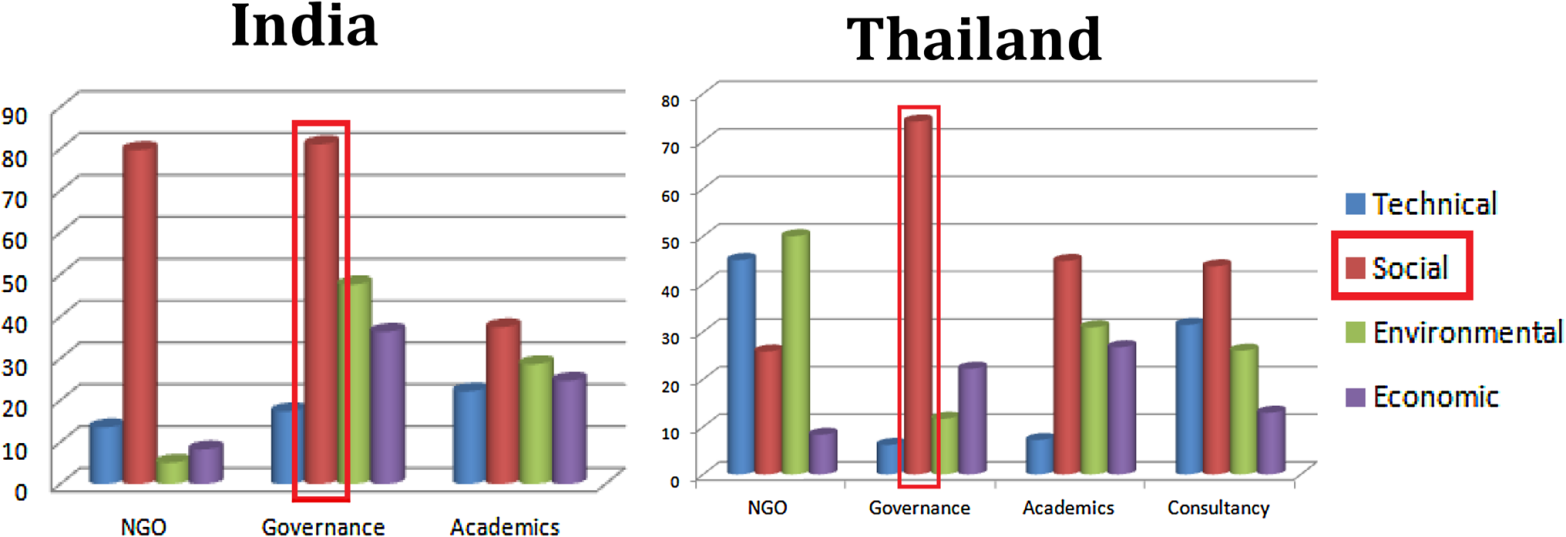
Appraisal diversity in assigning weights to each group of criteria for urban mobility case in India and Thailand

In the urban mobility cases in both countries, it was quite striking that it was the governance actors who assigned the highest importance to social sustainability of the emerging mobility trajectories (in India alongside NGOs). This is depicted in Fig. 6.

One qualitative substantiation of this result emerged when one of the participants, categorised as a governance perspective in India, explained that people will only prefer a mode of mobility if they think it matches with their status and position in the society. The criteria are thus closely linked with judgements concerning social and cultural perceptions and mind sets about the different forms of mobility. Under a criterion of community involvement, this participant also emphasised the importance of sufficient knowledge dissemination as a social criterion.

Looking carefully at the graph for the Thai Urban mobility case, (Fig. 6) it can be observed that there is a considerable difference across stakeholder groups, in the assignment of weights to what might be considered more ‘technical’ criteria in the framing of sustainability. These criteria typically included time predictability of mobility services and minimisation of travel time as well as energy efficiency, adaptive-ness and compatibility in energy systems. The governance actors and the researchers assigned very low weights to these technical issues, when compared to the consultants and NGO representatives.

**Fig. 7:**
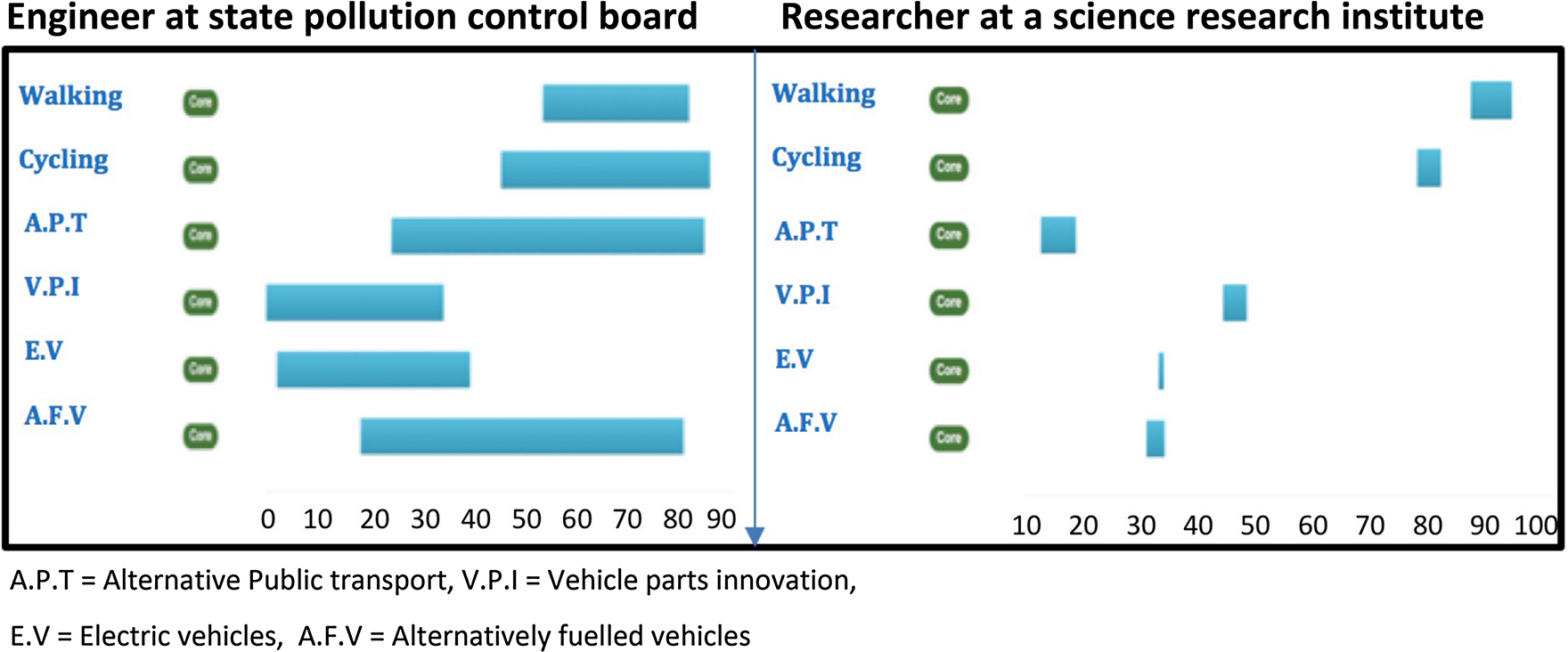
Appraisal diversity at an individual level for urban mobility case in India for an engineer at a state pollution control board and a researcher at a science and technology research institute

As mentioned in the beginning of this section, appraisal diversity also refers to the differences in ranking patterns at an individual level of contrasting viewpoints. Figure 7 demonstrates the comparison of the appraisals by two individual stakeholders for urban mobility in the Indian case. We can interpret that the engineer at a state pollution control board (left graph) was highly uncertain about sustainability of bus rapid transit (BRT) systems and CNG vehicles in spite of being overly optimistic about the fact that both are sustainable options. He was also optimistic about trajectories like Walking and Cycling and pessimistic about vehicle parts innovation like ultra capacitor and electric vehicles with moderate degree of uncertainty. In contrast to his appraisal, however, another stakeholder from a science technology and development research institute (right graph) expressed high optimism for sustainability of walking and cycling trajectories and pessimism for BRT—all with negligible amount of uncertainty.

The qualitative information collected in this MCM analysis tells us that appraisal diversity can also be identified if we look carefully into the ways in which each stakeholder perceived the scope and potential of each trajectory. For example, although the ‘solar city’ trajectory is considered a centralised system by some stakeholders, others consider this trajectory to be a ‘collection of technologies’, or even an ‘enabling environment to experiment with different solar technologies—each of which can be managed in small units’. From this point of view, solar city is a desirable option if there is a community or household ownership of individual applications constituting a large solar city project. This ‘if’ resulted in the especially diverse extreme ranges displayed in the scoring the trajectory. Some stakeholders argued that the solar city concept has great potential to address environmental sustainability issues, thus assigning high optimistic scores to this trajectory. Others explained that policy framings of this option are currently quite opaque, resulting in less confidence in more optimistic scenarios for the performance of this trajectory.

This section has demonstrated the importance of highlighting differences in criteria and uncertainties across social groups—as well as their associated patterns of reasoning. These may easily be missed in attending only to the aggregate picture in Sect. 4.1. The next section continues with differences in sustainability across different sectors.

### Sectoral diversity

**Fig. 8:**
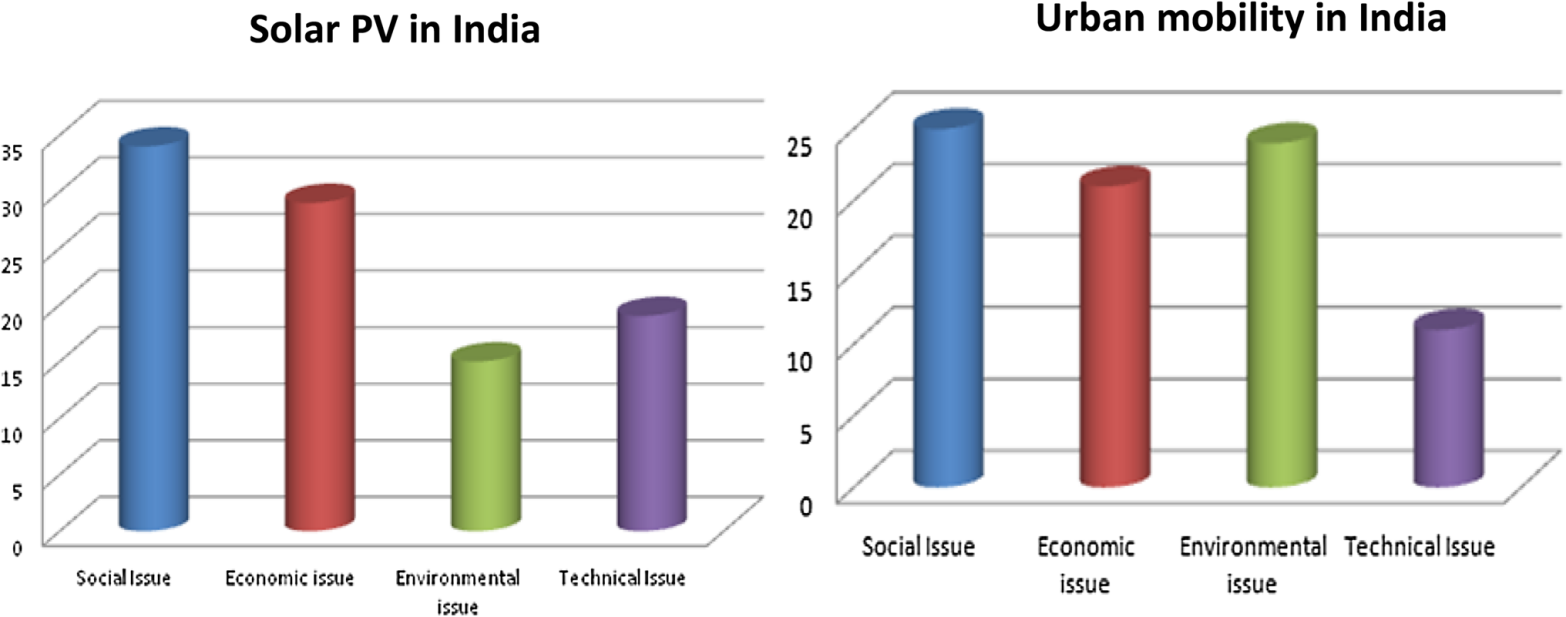
Sectoral diversity in number of criteria proposed for each group in solar PV and urban mobility in India

Diversity can also be observed across the two sectors studied in this research (energy through solar photovoltaic and urban mobility). This is evident, for instance, in respect of criteria, definitions and uncertainties as between degrees of optimism and pessimism. One of the striking differences between the two sectors is that criteria for environmental sustainability did not seem to be as important, either in numbers or in weights for the solar PV trajectories compared to urban mobility. Figure 8 (below) shows this diversity across the two sectors in India.

Here, we can observe that the number of criteria proposed to assess environmental sustainability of solar PV systems was significantly exceeded by the criteria proposed for social, economic or technical issue (the graph to the left). In contrast to this, for urban mobility (Fig. 8, graph to the right), environmental issue contains the second highest number of criteria, which follows after the highest number of criteria proposed for social issues of sustainability. In light also of associated qualitative findings, we can conclude that concerns over environmental sustainability were notably more pronounced and nuanced in the urban mobility sector cases in both countries.

The relatively low salience of environmental aspects of sustainability in the solar PV case in both countries can be interpreted in two ways. First, stakeholders suggesting environmental criteria in this case tended to assign relatively low weights to these criteria. Associated comments suggest that many of them simply assumed solar applications to be environmentally sustainable and, therefore, felt it more important to evaluate other (social, economic or technical sustainability) aspects more relevant to distinguishing between the relative merits of these trajectories. Second, many stakeholders mentioned just one or two environmental criteria, defining these such as to incorporate several environmental concerns in a single criterion. As an example of this, one stakeholder named her criterion in this case, ‘Reduction in environmental impact’. In the description of this single criterion, she talked about local air pollution, noise pollution, global climate mitigation strategies all the same time.

From a sectoral diversity point of view, this is an interesting observation, since such integration of several concerns in one criterion can only be seen in the solar PV cases. In the urban mobility cases, by contrast, criteria were much more reflective of specific environmental aspects of sustainability. Notwithstanding this overall pattern, it is all the more striking that a few stakeholders in the solar PV appraisal did raise specific concerns about provision for battery disposal for solar home systems, and use of agricultural land for construction of power plants. It can be concluded that even if the solar PV trajectories are perceived to be using fairly similar technologies, there were some instances when concerns arose over particular environmental issues under which options performed differently.

**Fig. 9:**
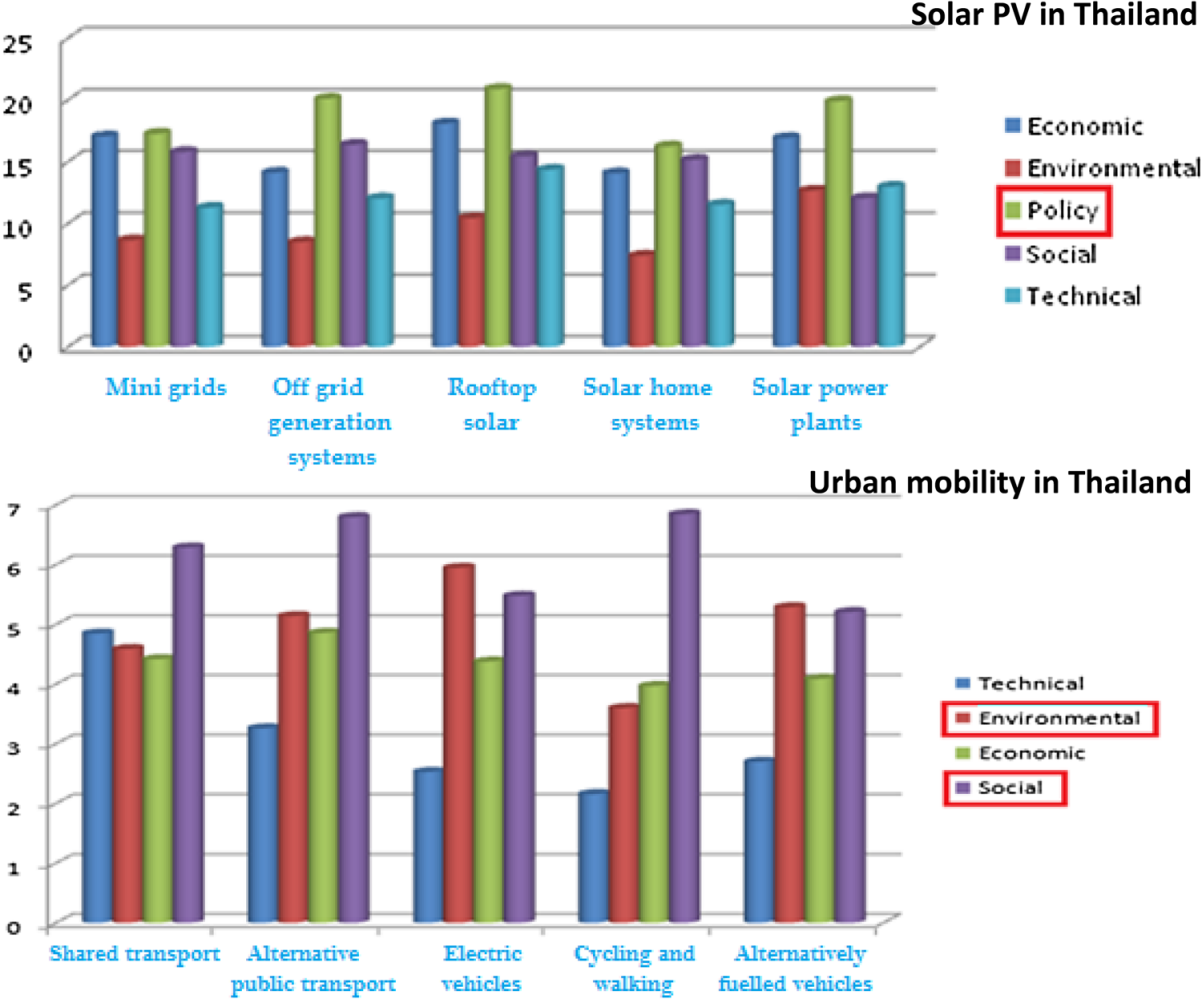
Sectoral diversity in the range of uncertainties for each trajectory in solar PV case and urban mobility case in Thailand

Diversity across the two sectors is also reflected in the expression of uncertainties, as illustrated in Fig. 9 for the two sectors in Thailand.

For the solar PV case (at the top), the highest range of uncertainties was expressed for policy-related criteria (rather than environmental, economic, social and technical criteria), while for urban mobility case (at the bottom), uncertainties were most prominent for social and environmental sustainability criteria. For mobility trajectories, none of the stakeholders even mentioned a supportive policy environment to be a relevant sustainability criterion. Instead, the stakeholders were more concerned about sustainability of urban mobility trajectories in terms of providing accessibility to all areas and to all people of the society and in terms of their capability to reduce pollution, congestion, emissions, etc. This difference, observed for two sectors in the same country, is intriguing because this implies that the stakeholders perceive that sustainability of solar PV systems is more dependent on enabling policy and governmental support than is the case for urban mobility systems.

In sum, this analysis of sectoral diversity demonstrates that even within the same country, the perception of sustainability differs markedly across energy and mobility sectors. Not only are the sustainability criteria and their respective weights different across the two sectors, but the ambiguities and uncertainties about the sustainability of the various trajectories also differ. Such diversity would have been less easy to observe, in a technique involving prior definition by the analyst of what constitutes ‘sustainability’. Finally, in the next section, we will turn to the diversity exhibited across the two countries.

### Geographical diversity

Geographical diversity concerns the contrasting difference in the appraisal results in the two case study countries, namely India and Thailand. One of the first observations in this regard can be presented in terms of the diversity of sustainability criteria expressed in the two countries. Affordability, for instance, is proposed as a crucial economic sustainability criterion by almost all the participants in India, while it is mentioned only once in Thailand. On the other hand, many stakeholders state safety issues as sustainability criteria in the urban mobility workshop in Thailand, but not much in India. These qualitative differences in type and frequency of the criteria proposed shows that, even while appraising the same types of trajectories-stakeholders in India and in Thailand, participants reflected upon their local and regional context and experiences and thereby set different priorities in ensuring sustainability of the systems.

**Fig. 10:**
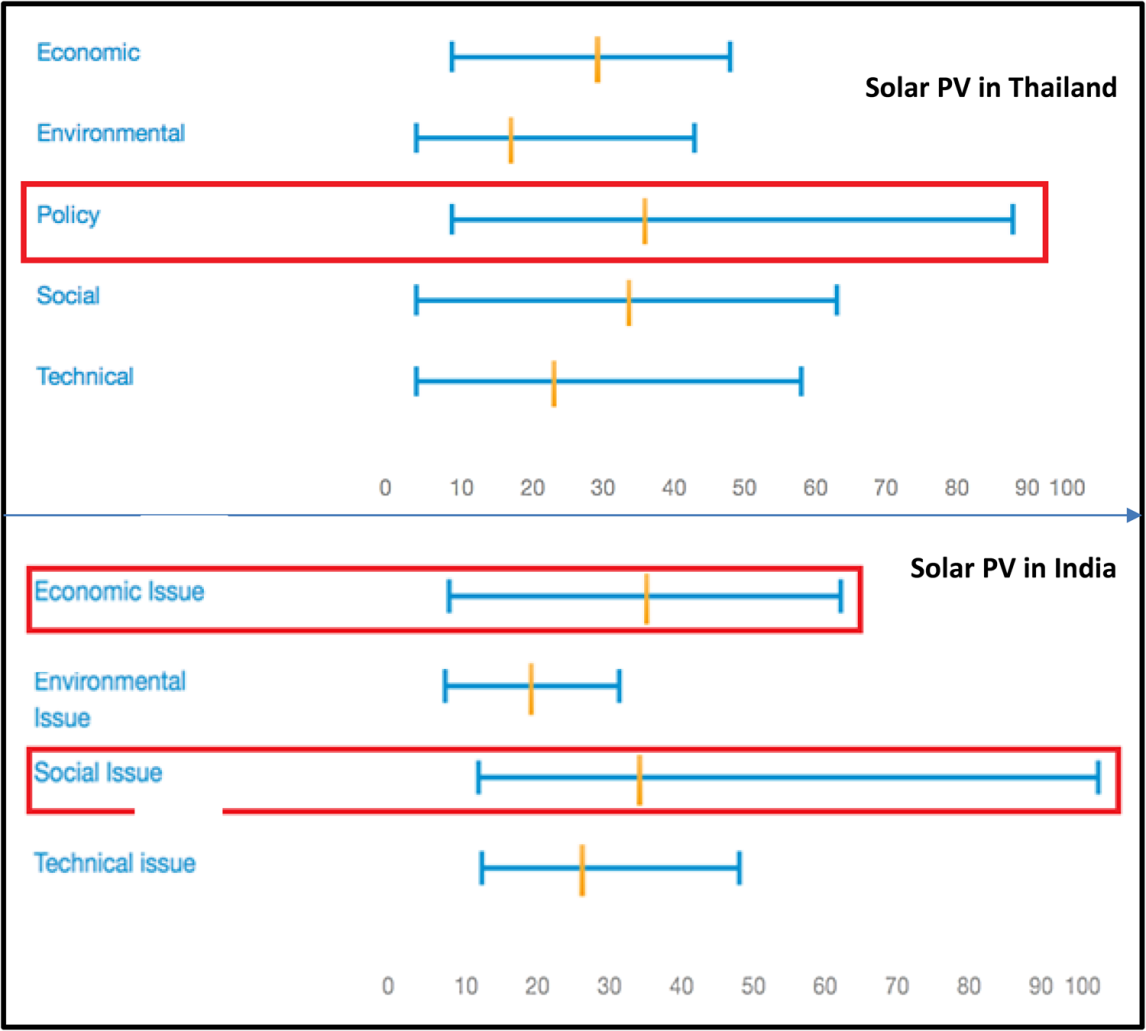
Geographical diversity in assigning weights to respective criteria groups (issues of sustainability) for solar PV appraisals in Thailand and India

Another notable geographical diversity for solar PV appraisal is that a far greater number of criteria related specifically to governmental support and policy incentives in Thailand than in India. Qualitative data in this regard justify distinction in Thailand but not in India, of a separate group of criteria under the heading of ‘policy’. In the appraisal of solar PV trajectories in Thailand, these policy criteria also received higher average weightings than did social, economic, environmental and technical sustainability criteria. This result is illustrated in Fig. 10, where the graph on the top represents the situation in Thailand, as compared to India in the bottom where participants rated social and economic issues of sustainability the highest.

**Fig. 11:**
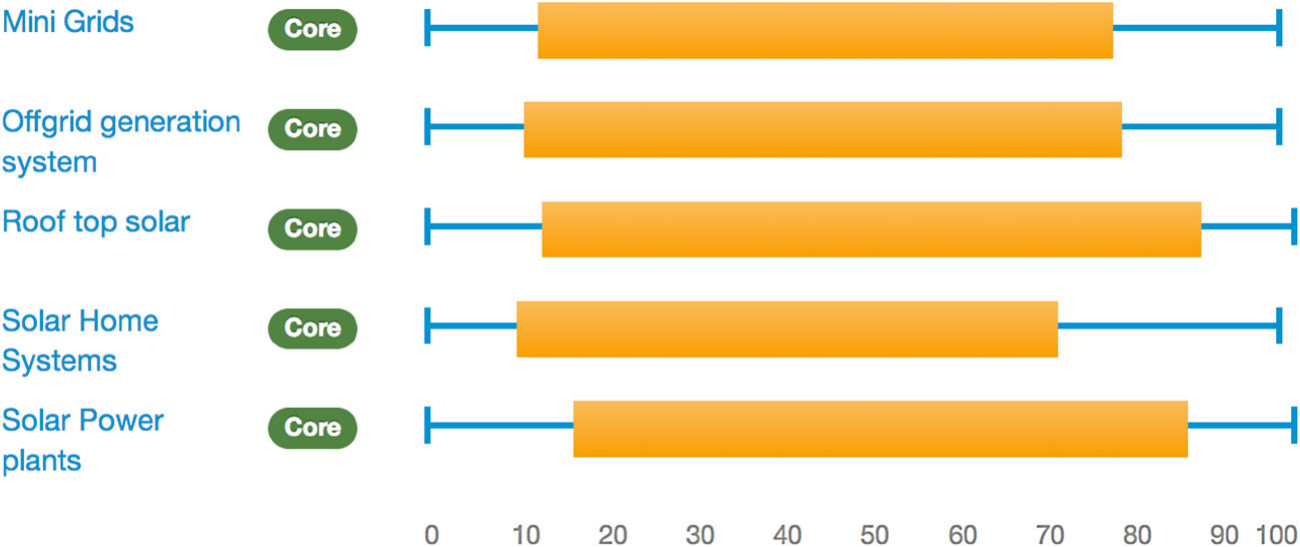
Performance diversity in solar PV case in Thailand

Following this assignment of highest significance to policy-related criteria in Thailand (but not India), it is perhaps relevant (despite major uncertainties) that Thai participants appraising solar PV tended also to express a discernibly stronger preference towards those solar trajectories that receive governmental policy and financial support. These trajectories (namely rooftop solar and solar power plants) were considered to be more sustainable options (in terms of higher optimistic scoring) in the final ranking of the trajectories. Figure 11 presents this result.

This trust and dependency on institutional policy and financial schemes seemed to be absent in the appraisal of solar PV trajectories in India, where the stakeholders were rather pessimistic and uncertain about sustainability of solar power plants in spite of supportive policy instruments like the National Solar mission in place. Here, they raised concerns over what were expressed in qualitative statements to be huge investment costs, long implementation times, transmission and distribution losses and land allocation requirements.

One of the other striking aspects of diversity between appraisal results in the two countries is in the levels of uncertainties with which the stakeholders appraised the trajectories. Relatively high levels of uncertainty can be observed in the appraisal of all trajectories for both solar PV and urban mobility systems in Thailand. Indeed, the high levels of uncertainty here contributed to a serious difficulty in interpreting aggregated performance diversity, in that it is difficult to see any clear overall difference in the sustainability performance across different trajectories (see Fig. 11, solar PV in Thailand). In the case of India, however, contrasting patterns of optimistic and pessimistic scoring contributed to a greater degree of confidence in interpreting the differences in sustainability appraisal of the different trajectories. (see Fig. 3 on solar PV in India).

**Fig. 12:**
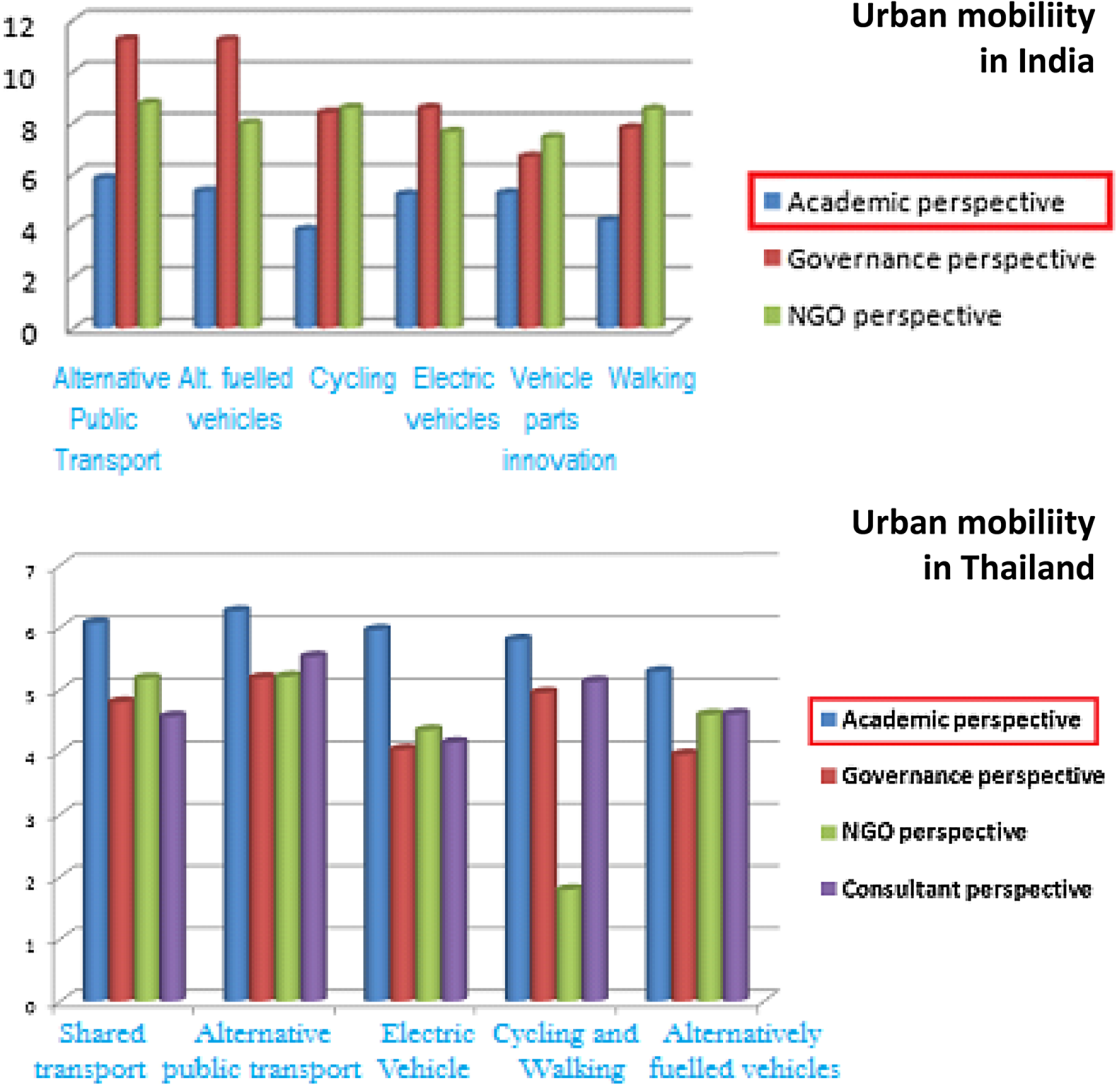
Geographical diversity in the range of uncertainties by each stakeholder group in urban mobility case in India and Thailand

Comparing the urban mobility cases for both countries, it can be observed that in India, stakeholders from an academic perspective expressed less uncertainty than other stakeholder groups. Interestingly, the opposite is true in Thailand, where academic stakeholders expressed the highest uncertainties when compared with other stakeholder groups in Thailand. This result is displayed in Fig. 12, where relatively short blue bars in the graph at the top represent the relative uncertainty level expressed by academicians in India, while the relative tall blue bars in the graph at the bottom represent the relative high levels of uncertainty expressed by Thai academics.

As with the other parameters analysed here, it would be hazardous to generalise to an entire country, the differences in pictures interpreted here as geographical diversity. Any such analysis would need to be based on more detailed analysis of the qualitative data to substantiate the extent to which divergent cultural factors may or may not be implicated. For the purpose of simply documenting the potential salience of diversity, however, this evidence serves quite well. Given the overall similarities in the final rankings displayed by the different experimentation trajectories in the two countries, it is quite striking that the underlying perceptions of sustainability and the specific ways in which these trajectories are appraised (optimistic, pessimistic views, expression of uncertainties and ambiguities) are so contrasting between the two countries. Following results in other MCM studies (Burgess et al. 2007), this underscores the importance of not over-interpreting the practical policy implications of wide discursive differences, and not over-interpreting any similarities in practical policy implications as indicating wider contextual similarities. Either way, it appears that diversity of many kinds remains a crucial factor to analyse.

## Conclusion

This paper started with the ambition of contributing to debates around questions of what sustainability means, to whom, in what contexts, and with which kind of implications. Building upon extensive research in India and Thailand, this paper asked the question: How are emerging innovation trajectories for solar energy and urban mobility appraised by various actors under different perspectives in India and Thailand? The following conclusions can now be drawn.

As a preface, we note that the current approach has of course only taken a snapshot in time. It is reasonable to assume that sustainability appraisal may be expected not only to be highly context specific, but also temporally specific. This can especially be expected for rapidly industrialising parts of the global south where the quest for sustainability transitions is tied up with other far-reaching processes of societal transformation. So conclusions must be cautious and qualified concerning the generalisability of any specific patterns noted here.

This said, the first conclusion to draw is that this paper mobilised an innovative methodology and developed a novel pragmatic framework for unpacking sustainability in terms of performance, appraisal, sectors and geography. The application of this framework to solar PV and urban mobility experiments in India and Thailand demonstrates a vast degree of diversity in terms of criteria, uncertainties and rankings across different sectoral and national contexts and between different social groups and individuals. The magnitude and pervasiveness of these diversities remain a crucial issue, irrespective of any questions that may be raised about the particularities of the individual findings or the completeness or representativeness of the appraisal as a whole.

Simply in their own right, the existence of such diversities implies that those involved in transition analysis must be extremely cautious in assuming any objective status for the ‘sustainability’ of sustainability experiments on the basis of more conventional approaches such as integrated assessment that simply calculate and rank options. Moreover, which ‘niches’ or ‘cases’ to choose for analysis is not a neutral choice, but is evidently highly normative, and deserving of considerable further reflexive thought. For instance, our analysis demonstrated that participants in the present appraisal exercise highlighted a sustainability preference for slower forms of mobility, such as walking and cycling—cases which are rather unconventional empirical domains in the study of sustainability transitions. In parallel, cases that have been studied more in-depth such as cleaner transport fuels were received with more reservation.

Second, our research suggests that even within apparently singular socio-technical fields, there exists a high degree of uncertainty and ambiguity concerning future sustainability performance. For instance, the ranking ranges are massively overlapping for all socio-technical options considered in relation to solar PV. Hence, whether decentralised options such as lighting or roof top systems or solar home systems are more or less sustainable than centralised options, such as large-scale power plants or solar cities, is highly uncertain and dependent on assumptions and perspectives concerning the unfolding of particular socio-technical configurations as well as possible future conditions. For the study of transitions, this implies that research could engage more with unpacking these uncertainties and diverse possible socio-technical configurations, even within (apparently) singular technological fields. Again, this finding applies without any need to claim completeness or representativeness for the particular perspectives engaged here.

Third, the analysis of what is here called appraisal diversity demonstrated a high degree of diversity in the kinds of criteria and levels of uncertainty displayed across contrasting social perspectives as well as different individuals. Perhaps more important is the qualitatively informed finding that diversity in criteria and uncertainty persist when comparisons span sectoral and national contexts. The implication is that what sustainability means, how it should be assessed, and with what kind of implications, is very much context-dependent. This research, for instance, showed that criteria relating to policy conditions were held to be crucially important for nearly all participants in Thailand, whilst these kinds of criteria were hardly mentioned by participants of any kind in India. Whilst not exploring these reasons empirically here (which may have to do with the differences in political regimes in India and Thailand), the broader implication is that the study of sustainability transitions must be addressed within specific geographical contexts. Whilst national boundaries have been taken for granted as key spatial level of analysis, future research must explore empirically what the relevant special scales for unpacking sustainabilities are (Raven et al. 2012).

Fourthly—and perhaps most importantly—it follows from the present analysis of these different kinds of diversity, that sustainability in any practical policy context like those addressed here—is a significantly more political matter than is typically conceded in many kinds of academic and policy analysis in this field (Leach et al. 2010). Where appraisal tends to deliver results to policy making that assert singular (apparently prescriptive) pictures of the relative performance of different options for action, then it can have the effect of ‘closing down’ appreciation for the kinds of uncertainties, ambiguities and variabilities documented here (Stirling 2008). Where these are not deliberately illuminated in analysis, their existence will remain correspondingly neglected in policy—and vulnerabilities are exacerbated to strategic behaviour in the design or implementation of analysis.

The crucial question that arises then, in this regard, is about how in the light of all these kinds of diversity, policy actors can reasonably proceed to make decisions on crucial matters like the sustainability of energy or transport infrastructures.^6^ The answer here lies in the qualities of humility and reflexivity in appraisal: a willingness to acknowledge that the policy interventions are typically not justifiable purely by means of analysis (Stirling 2006). It will almost always be the case in complex field like those addressed here that value judgements and other subjectivities will also play determining roles. In this respect, a method like MCM has the virtue that it is rigorous not only about contrasting technical understandings and their respective uncertainties, but also about divergent political and normative positions. By presenting appraisal results in plural and conditional (rather than unitary and prescriptive) ways (Stirling 2010), a ‘mapping’ method like MCM arguably allows not only enhanced rigor in the illumination of these unavoidable dilemmas, but also greater democratic accountability and social robustness in the justification of resulting decisions, and as such may have high relevance for policy making. Decisions can still be made, but must be justified as much in relation to explicit evaluative perspectives, as to ostensibly technical analysis. With worldwide political trends increasingly challenging the role of democracy in decision making, this attribute is arguably becoming increasingly salient.

## Footnotes

1 See for instance relevant contributions in Sustainability Science, such as frameworks to assess sustainability of water governance alternatives (Kuzdas et al. 2016), livelihoods (Veisi et al. 2014), urban systems (González-Mejía et al. 2014), entire regions (Hara et al. 2009), intra-regional partnerships (McLarty et al. 2014), food systems (Cochran et al. 2016), policy driven agricultural practices (Purushothaman et al. 2013) and agricultural systems such as rice farming (Roy et al. 2014), wheat-based cropping system (Moeller et al. 2014), and irrigated commercial maize production (Bausch et al. 2014).

2 Eames and McDowall followed the Transition Management approach (Loorbach 2010), which implies the development of visions, followed by the appraisal of these visions in multi-stakeholder dialogues. In contrast, the current paper takes outset in the literature around Strategic Niche Management (Kemp et al. 1998), which emphasises aligning expectations on the basis of ongoing experimentation. Hence, the identification of options for appraisal took place on the basis of a newly constructed database of ongoing sustainability experiments rather than envisioned futures.

3 For Thai solar PV initiatives we have used the official Energy Regulatory Commission website as of January 2012: http://www.erc.or.th/ERCWeb2/Default.aspx.

4 See for instance Ghosh (2014), Wieczorek et al. (2015), Sengers (2016) and Jolly (2016).

5 Prioritising faithful attention to multiple stakeholder’s own perspectives on complex contentious issues in science and technology, this new method systematically captures qualitative and quantitative information concerning alternative framings and evaluative priorities across a range of different options, and illuminates the variety of different appraisals that arise.

6 We thank one of the reviewers for asking these relevant questions.
